# Supplementary material for: Establishment of mouse model of inherited PIGO deficiency and therapeutic potential of AAV-based gene therapy
Source: Nat Commun. 2022 Jun 3;13:3107. doi: 10.1038/s41467-022-30847-x (PMC9166810; doi:10.1038/s41467-022-30847-x)
Supplement: Supplementary file 1 — Supplemantary information [file 41467_2022_30847_MOESM1_ESM.pdf]

Supplementary information

Title

Establishment of mouse model of inherited *PIGO* deficiency and therapeutic potential of AAV-based gene therapy

Ryoko Kuwayama, Keiichiro Suzuki, Jun Nakamura, Emi Aizawa, Yoshichika Yoshioka, Masahito Ikawa, Shin Nabatame, Ken-ichi Inoue, Yoshiari Shimmyo, Keiichi Ozono, Taroh Kinoshita, Yoshiko Murakami

Supplementary Figure 1-12

Supplementary Table 1-2

Supplementary Figure 1

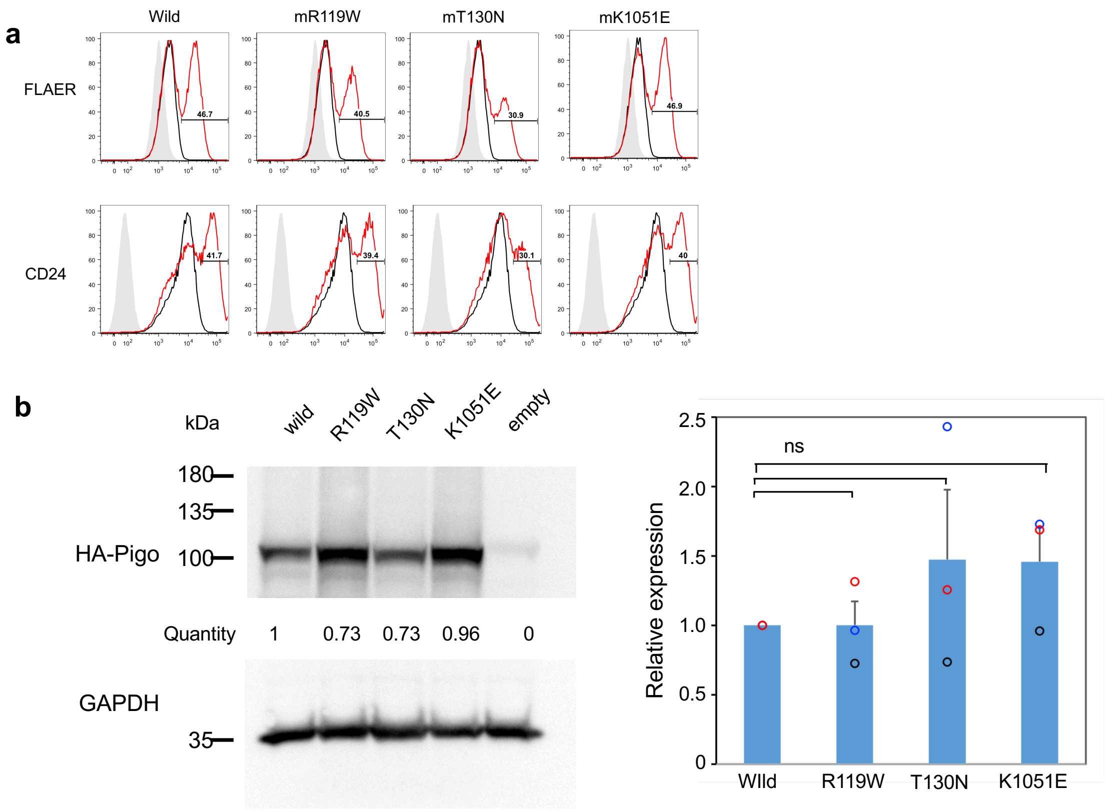

Flow cytometry and Pigo protein expression of *Pigo*-knockout Neuro2a cells transfected with wild-type *Pigo* cDNA or three types of mutant cDNA. a. (From left to right; wild, Arg119Trp (R119W), Thr130Asn (T130N), Lys1051Glu (K1051E)). Defective GPI-AP expression, stained by FLAER in the upper panel, and CD24 expression in the lower panel of *Pigo*-knockout Neuro2a cells were partially restored by transfection of each mutant cDNA. Shadows, isotype controls; black lines, *Pigo*-knockout Neuro2a; red lines, various missense mutants driven by the strong SRα promoter. The highest percentage of restoration was 46.9% (FLAER) and 40% (CD24) in K1051E, followed by R119W (40.5%, 39.4%), T130N (30.9%, 30.1%). **b.** Lysates of the transfectants shown in **a** were subjected to SDS PAGE and immunoblotting for HA tag. For quantification, relative protein expression was determined by dividing HA-Pigo band intensities by those of GAPDH and by Luciferase activity to normalize for both loading and transfection efficiencies and resulting values converted to make the wild type value as 1. The graph shows the results from the three independent experiments. Data are presented as mean values + SEM. (t.test, two-sided) Source data are provided as a Source Data file.

Supplementary Figure 2

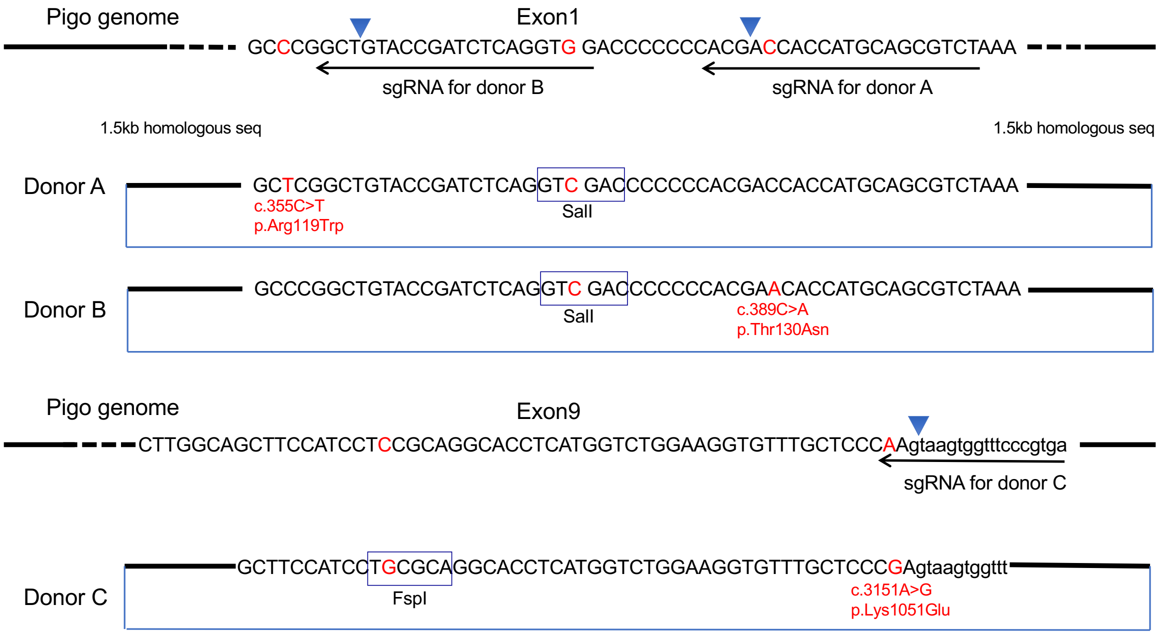

Structure of sgRNAs and donor vectors for generating each line of *Pigo*-KI ES cells. For A-line, B-line and C-line KI cells, ES cells were co-transfected with Cas9 expressing vector and the donor A or B or C (pBluescript), respectively. Arrow heads, cleavage sites of Cas9.

Supplementary Figure 3

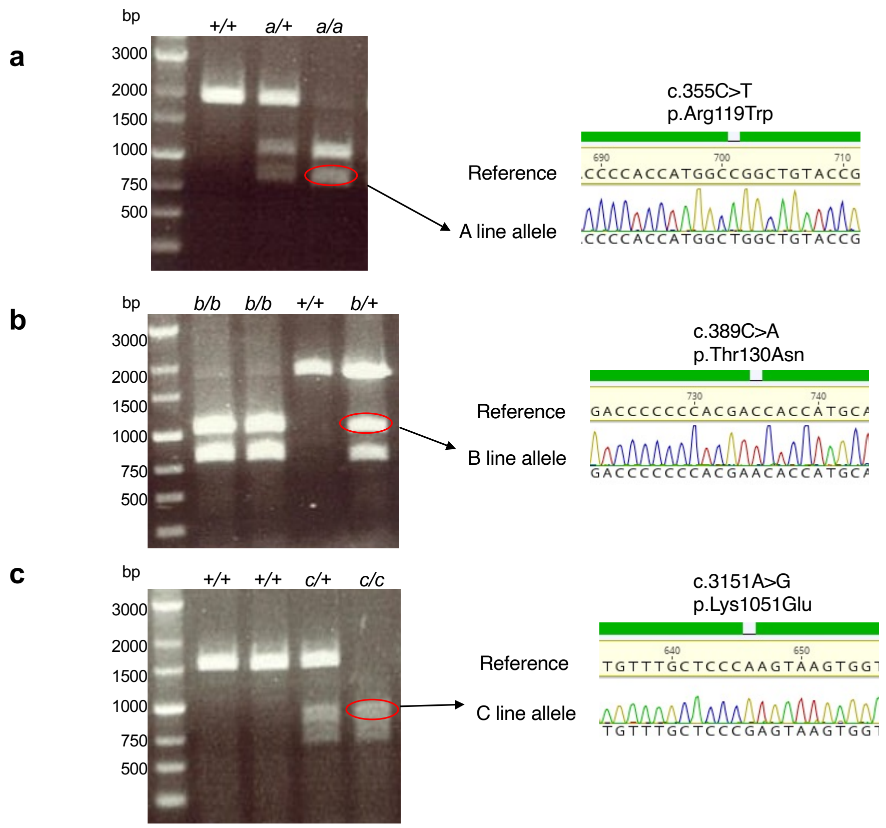

Sanger sequences of PCR fragments amplified from the tail DNA of three mutant mice. a and b. PCR products amplified from A-line and B-line mice genomic DNA were cut with SalI and the corresponding fragments were sequenced. **c.** PCR products amplified from C-line mouse genomic DNA was cut with FspI and the corresponding fragment was sequenced. These experiments were repeated at least three times.

Supplementary Figure 4

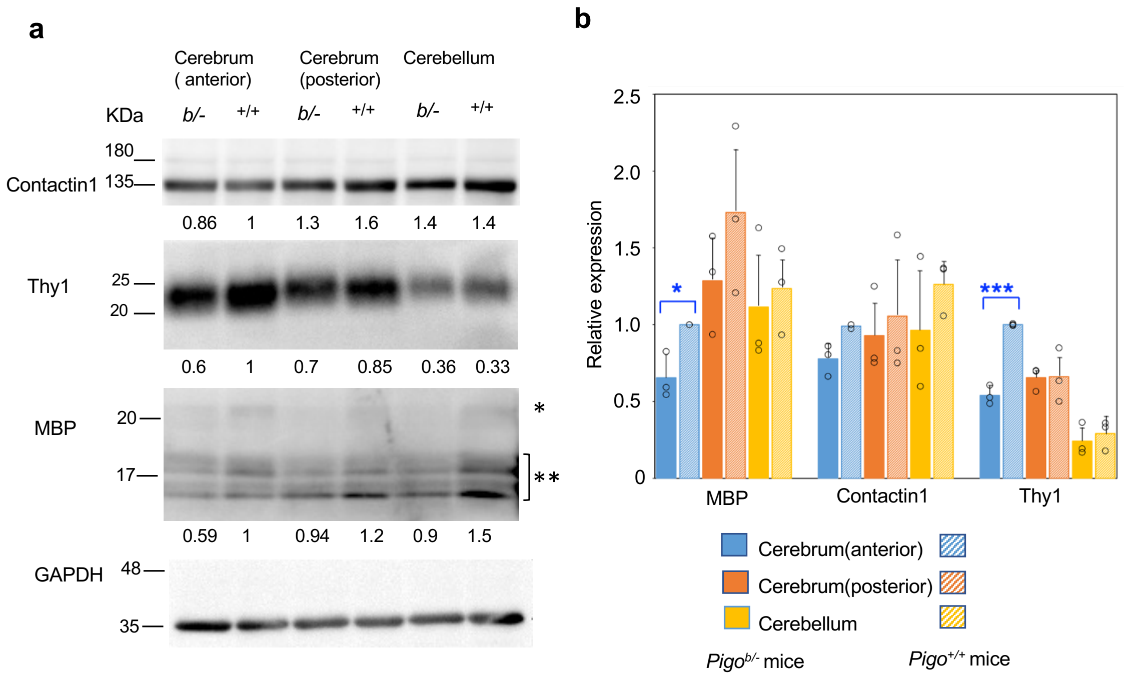

Expression of GPI-APs in the brain of *Pigo*$^{b/-}$ mice. a. The representative data of western blot analysis of *Pigo*$^{b/-}$ mouse brain. Expression of Thy1 in *Pigo*$^{b/-}$ mouse was decreased to about 60-82% of the control, whereas the expression of contactin1 was not decreased. Myelin basic protein (MBP, combined * and **) was also decreased to 58-80% of the control, suggesting that myelination is affected in *Pigo*$^{b/-}$ mouse. Quantity, band intensity of each protein was normalized with that of GAPDH and converted the wild type (+/+) as 1. **b.** Combined quantitative analysis from three pairs of *Pigo*$^{b/-}$ and *Pigo*$^{+/+}$ mice. MBP and Thy1 proteins were decreased in *Pigo*$^{b/-}$ mice (*; p<0.05, ***; p<0.001; t.test, two-sided; P value, MBP, 0.016; Thy1, 2x10^{-4}) Mean+ SD for relative expression of each protein is shown. Source data are provided as a Source Data file.

Supplementary Figure 5

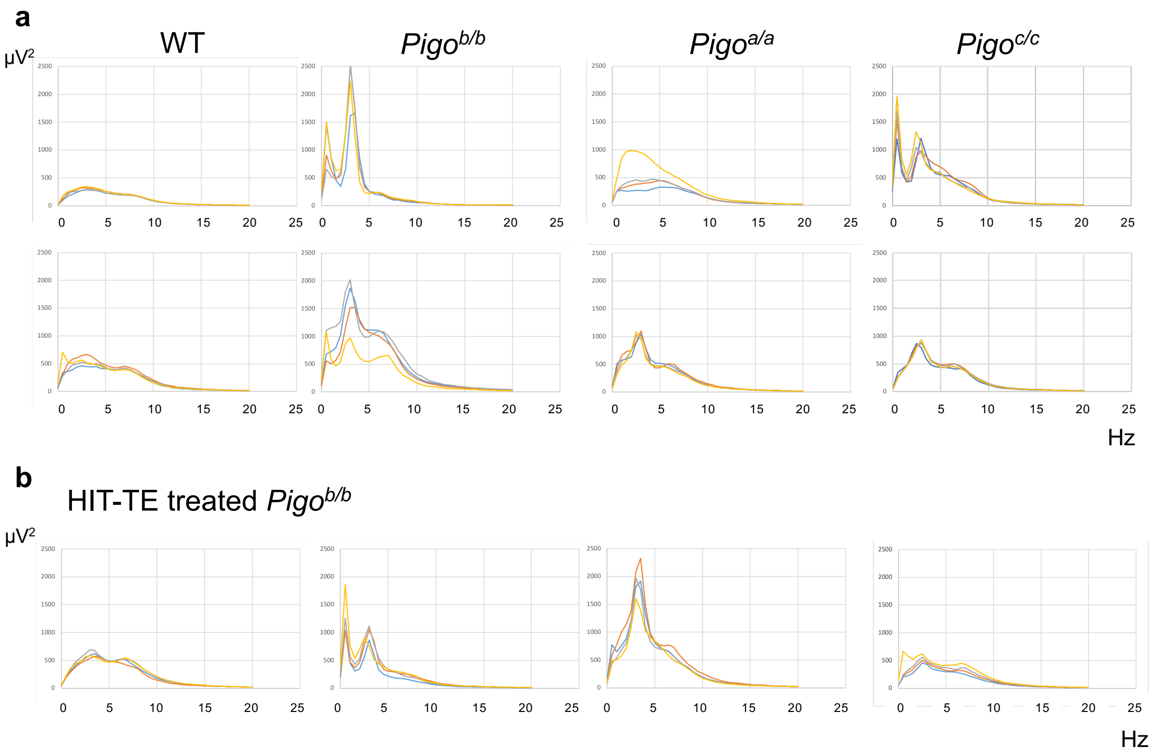

Fast Fourier transform (FFT) power spectral analysis of EEG. a. Power spectrum of average FFT of 8-hour recording in dark phase, showing data of two mice in each line. **b.** Power spectrum of average FFT of four HITI-TE-treated *Pigo*$^{b/b}$ mice. Colored lines show FFT spectrum of each two-hour recording of EEG within 8 hours in total.

Supplementary Figure 6

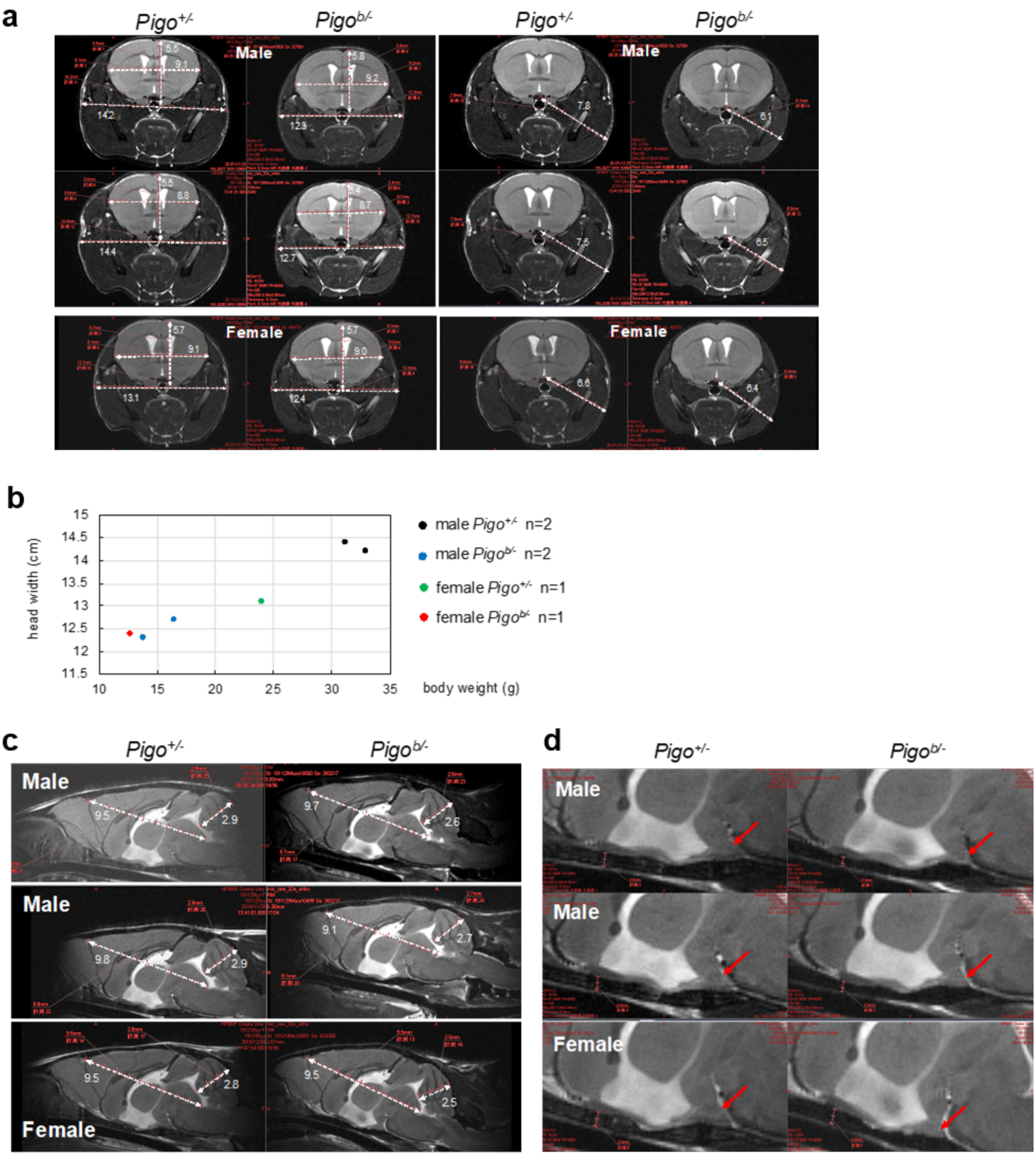

In vivo T_2 weighted MRI of mouse head and image analyses. a. *Pigo*$^{b/-}$ mice commonly showed decreased volume of the skeletal muscle on the heads (82% of control). **b.** The body weight of the mice was correlated with their head width. n, number of animals. **c.** The volumes of the cerebellum were slightly decreased (90% of control). **d.** The pituitary glands were smaller in *Pigo*$^{b/-}$ mice than those in their littermates.

Supplementary Figure 7

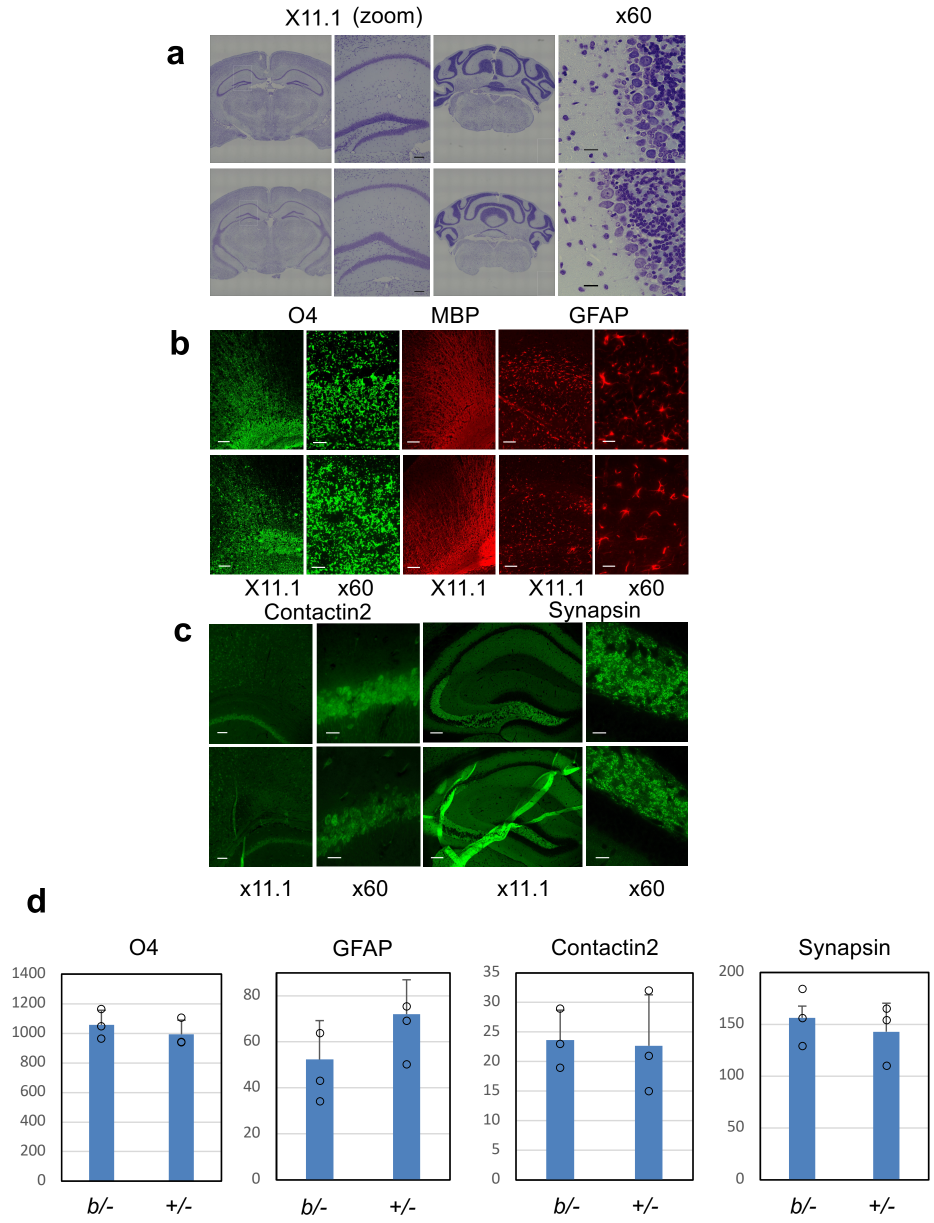

Histological analysis of the brain of *Pigo*$^{b/-}$ mice. a, Nissle staining of the brain from the 4-month-old *Pigo*$^{b/-}$ mouse showing no abnormalities in the *Pigo*$^{b/-}$ mouse including Purkinje cells (right end panels). Upper panels, *Pigo*$^{+/-}$ mouse; lower panels, *Pigo*$^{b/-}$ mouse. This is the representative data from two pairs of mice.

b. and c. Immunohistochemical staining of the brain of 4-month-old *Pigo*$^{b/-}$ mouse showing no abnormalities. Upper panels, *Pigo*$^{+/-}$ mouse; lower panels, *Pigo*$^{b/-}$ mouse. O4, a marker for the oligodendroglia; MBP, myelin basic protein; GFAP, a marker for the astrocyte. Scale bars in x11.1, 100 μ m; scale bars in x60, 20 μ m This is the representative data from two pairs of mice. **d.** Quantified data from b. and c. (x60); Average of area (for O4), cell numbers (for GFAP and Contactin2) and dot numbers (for Synapsin) were calculated in three parts of the brain tissue from each mouse. Data are presented as mean values + SD. Source Data are provided as a Source Data file.

Supplementary Figure 8

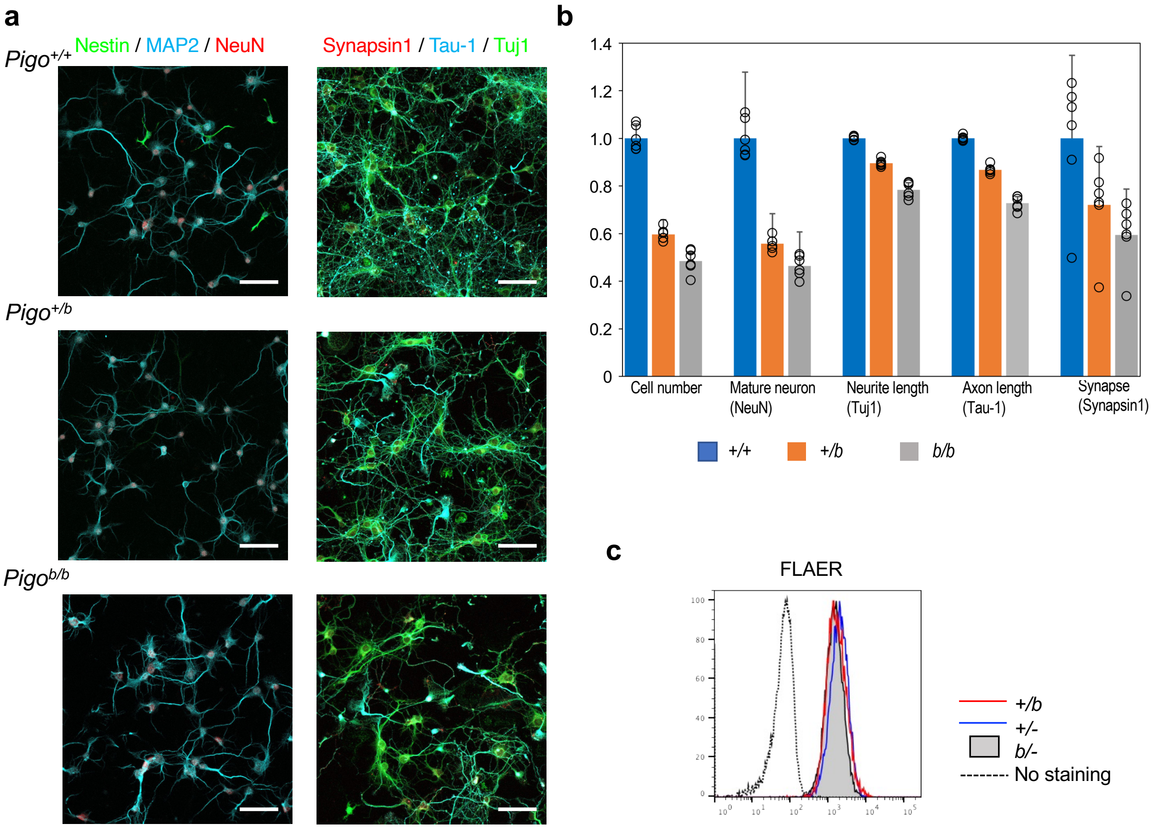

Primary culture of the neurons and embryonic fibroblasts from *Pigo*$^{b/b}$ or *Pigo*$^{b/-}$ mice a. After 7-day culture, neurons were stained with various antibodies. NeuN for mature neurons; Tuj1 for neurites; Tau-1 for axons; Synapsin1 for synapses. Scale bars represent 50μm. **b.** The graph showed the quantitative analysis of **a**. Vertical axis indicates counted numbers or length of each category, converted the wild type (+/+) as 1. Mean+SD for each is shown. This is a result of six independent cultures. **c.** FACS analysis of the embryonic fibroblasts established from the *Pigo*$^{b/-}$ mouse and its littermate control mice stained for GPI-APs with fluorescent-labeled inactive toxin aerolysin (FLAER).

Supplementary Figure 9

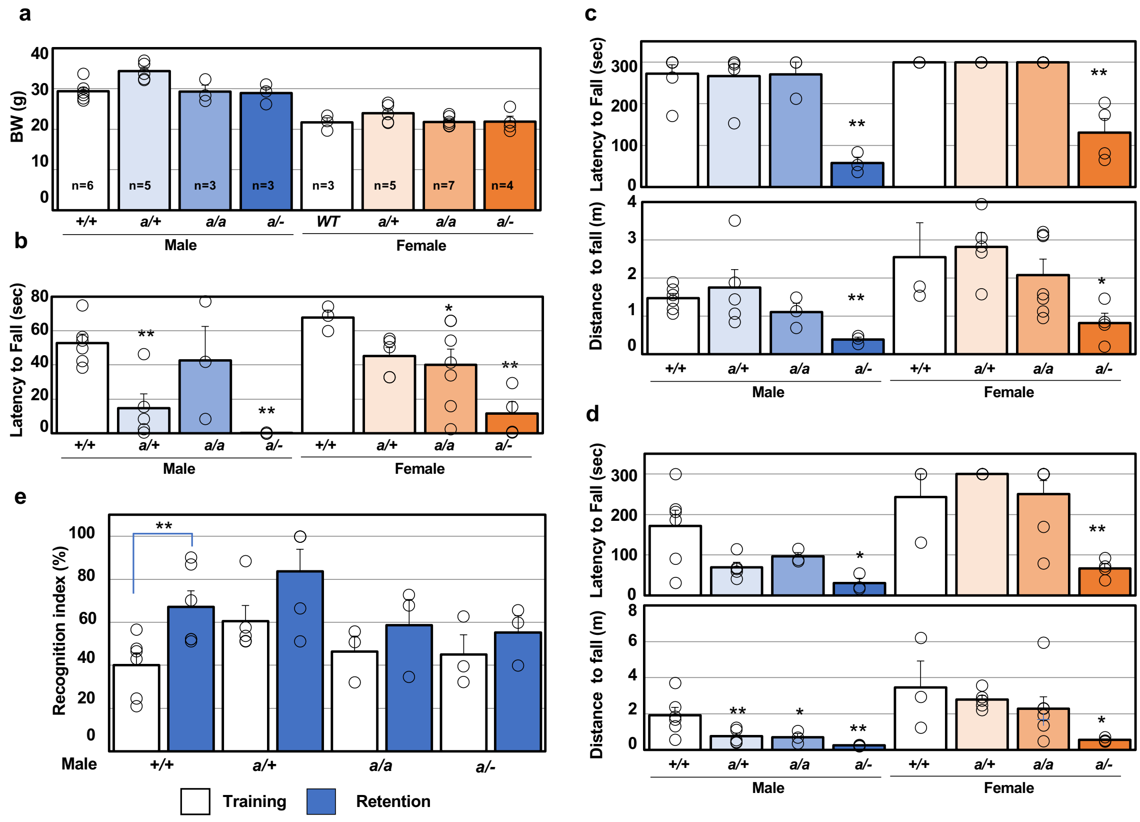

Behavioral tests of *Pigo* A-line KI mice. a, The average body weight of monitored mice. Male and female mice include four groups: *Pigo*$^{+/+}$, *Pigo*$^{a/a}$, *Pigo* $^{a/+}$, *Pigo*$^{a/-}$. No significant differences in body weight were observed between groups. **b,** Rotarod tests. **c,** Four limb hanging test (11mm). **d,** Four limb hanging test (5mm). The latency to fall (the upper figure), the distance traveled (the lower figure). **e,** Novel object recognition test. Only male wild mice spent more time exploring novel objects than the familiar objects with statistical significance. The number of each group of mice (**a-d**) was shown in **a**. Data are presented as mean values + SD. (t.test, two-sided)

Supplementary Figure 10

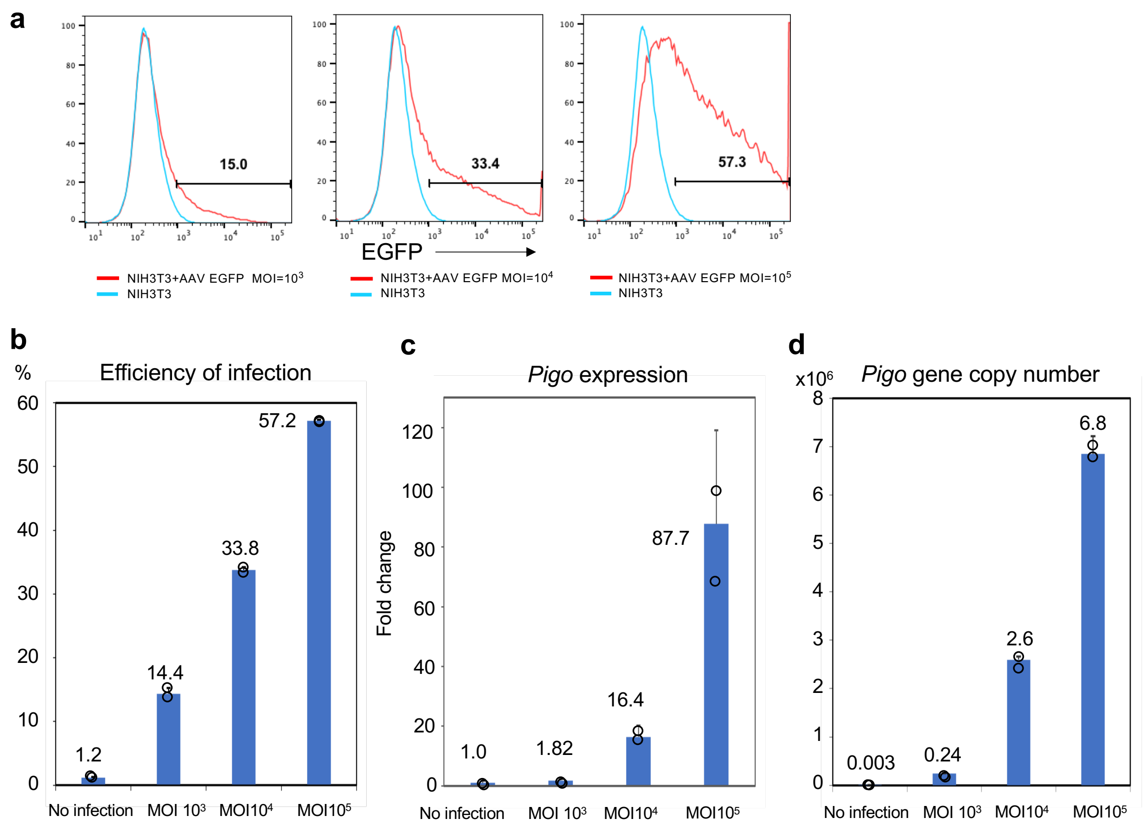

Comparison of the ITR driven *Pigo* expression with the endogenous *Pigo* expression in NIH3T3 cells a. FACS analysis of NIH3T3 cells infected at different MOI with AAV-PHP.eB bearing nEF-driven EGFP. Representative data from two independent experiments. **b.** Infection efficiencies calculated from data from two independent experiments shown in **a. c.** *Pigo* mRNA levels in the infected cells at different MOI. Endogenous level is set to 1. Triplicate analysis from two independent experiments. **d.** *Pigo* gene copy numbers in the non-infected cells and the infected cells at different MOI. Triplicate analysis from two independent experiments. Data are presented as mean values + SD. Source data are provided as a Source Data file.

Supplementary Figure 11

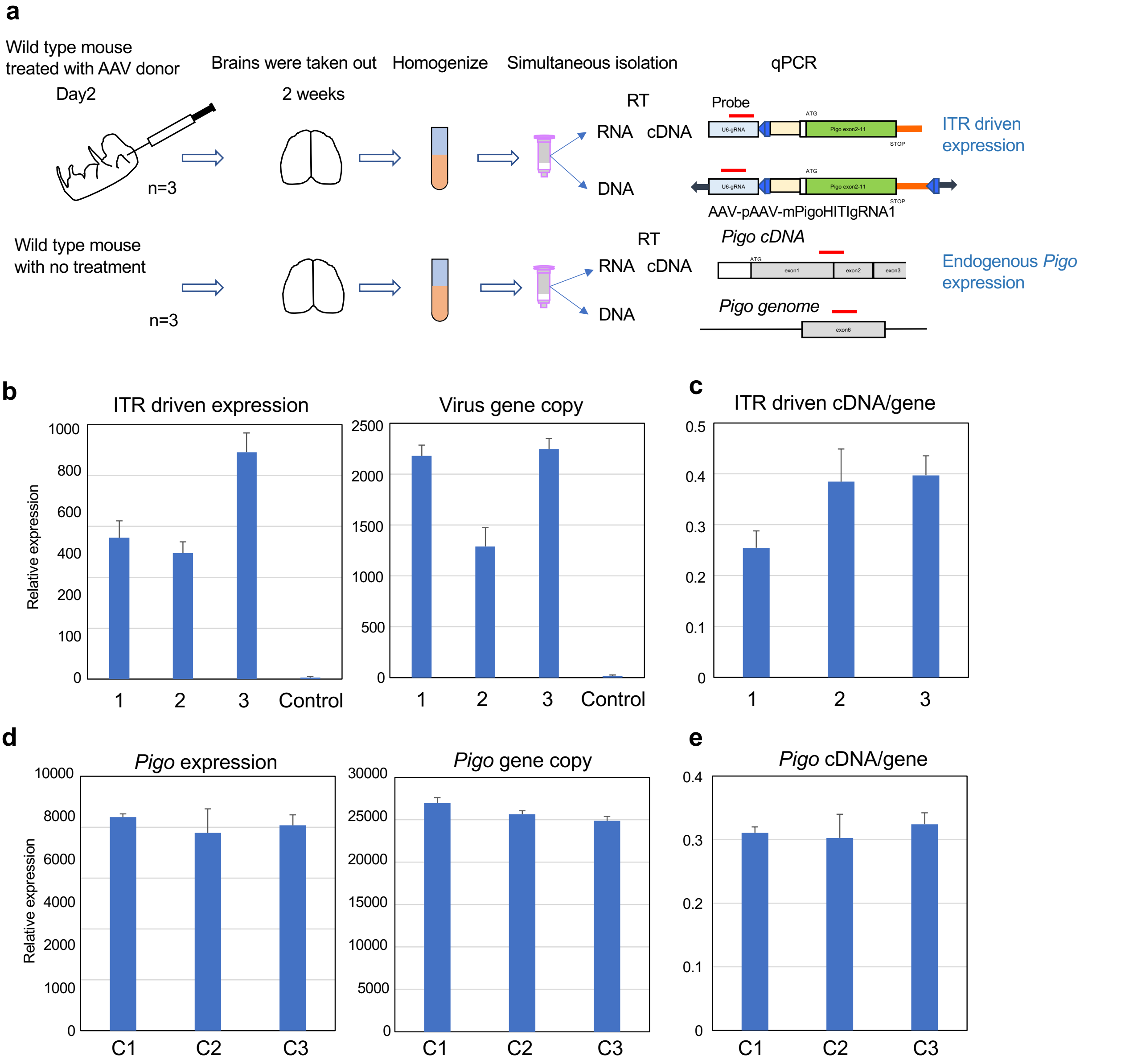

Comparison of the ITR driven Pigo expression with the endogenous *Pigo* expression in the mouse brain. **a.** Scheme of the experimental procedure. (See method section) **b.**ITR-driven *Pigo* cDNA expression (left) and AAV derived *Pigo* gene copy numbers (right) in the whole cerebrum from three mice (1-3) treated with AAV donor. Control is a non-treated littermate. The data were from the triplicate analysis. **c.** Relative ITR-driven cDNA expression per gene in the AAV donor treated mice. **d.** Relative endogenous *Pigo* cDNA expression (left) and *Pigo* gene copy numbers (right) in the whole cerebrums from non-treated mice (C1-C3). The data were from the triplicate analysis. **e.** Relative endogenous *Pigo* cDNA expression per gene in the non-treated mice. Data are presented as mean values + SD. Source data are provided as a Source Data file.

Supplementary Figure 12

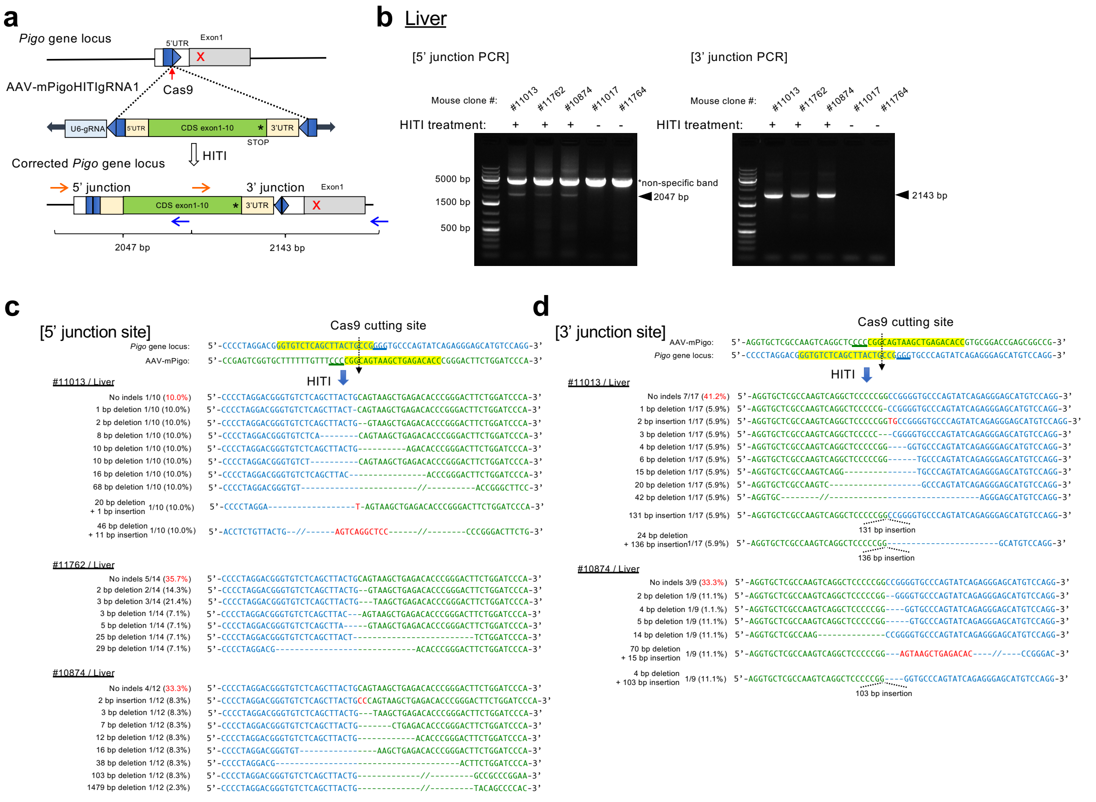

HITI-mediated genome editing in the liver via systemic injection in *Pigo*$^{b/b}$ mice.

a, Schematic representation of the *Pigo* KI allele b (T130N, c.389C>A) genome editing by a HITI donor. After genome editing mediated by NHEJ-mediated HITI, the *Pigo* full-coding cDNA including corrected mutation are inserted in 5'UTR, upstream of mutated exon 1. Blue pentagon, Pigo exon 1 gRNA target sequence in *Pigo* 5'UTR. Red arrow or black line within blue pentagon, Cas9 cleavage sites. Red and blue arrows, PCR primers.

b, Validation of correct genome editing by genomic PCR in the liver. Asterisk shows non-specific band. These experiments were repeated at least three times. **c,** Sequencing analyses of 5' junctions of the integration sites in the liver of HITI-TE-treated *Pigo*$^{b/b}$ mice.

d, Sequencing analyses of 3' junctions of the integration sites in the liver of HITI-TE-treated *Pigo*$^{b/b}$ mice.

Supplementary Table 1

New PTZ scale referring to modified Racine scale

Score	Behavioral stage	EEG findings
0	Normal	Wake rhythm
1	Normal	Spike-wave discharges
2	Whisker trembling, sudden behavioral arrest, facial jerking	EEG slowing, increased amptitude, intermittent spike-wave discharges
3	Neck jerks	Sharp spikes, followed by spike-wave discharges
4	Clonic seizures (sitting)	High frequency, small amplitude rhythmic waves
5	Clonic, tonic-clonic seizure (lying on belly)	High-amplitude polyspikes, spike-wave discharges
6	Clonic, tonic-clonic seizure (lying on side) & wild jumping	High-amplitude polyspikes, spike-wave discharges
7	Tonic extension, possibly leading to respiratory arrest and death	EEG trace appears almost flat

Supplementary Table 2

List of primers used in this study

Primers	Sequence
primer 1	5’-TTGCCACCCTGGAAATGTTG-3’
primer 2	5’-TAGAGGTGTTCCAAGATGCCG-3’
primer 3	5’-CCGGCTCAGAGTTTTCTCATT-3’
primer 4	5’-GAAACATAGTGCTTCAAACTGTG-3’
primer 5	5’-GAAAGGAGCGGGCGCTAGGG-3’
primer 10(5’RACE)	5’-GATTACGCCAAGCTTACATCCCACGAACCGCCGTCCAGGGTAG-3’
mPigoHITI5’-F1	5’-TGGTCCCTGGGCTTTTCTTCTTTCATGCTT-3’
mPigoHITI5’-R1	5’-AGGTATGAAGAAACCGGGACACCTGCT-3’
mPigoHITI5’-F2	5’-CGAGCCCTGCCGCTGCACTTCCG-3’
mPigoHITI5’-R2	5’-ACATCCCACGAACCGCCGTCCAGGGTAG-3’
mPigoHITI3’-F1	5’-ATGGTCTGGAAGGTGTTTGCTCCCAAGTTCA-3’
mPigoHITI3’-R1	5’-GAACCCGGCCAACACCTGGACATGC-3’
qPCRmPigo-F1	5’- GCAGTAACTTTGCCAGCCATGC-3’
qPCRmPigo-R1	5’-CACTGTGTGCAGGTCTCTGAC-3’
qPCRmTbp-F1	5’-TATGACCCCTATCACTTCTG-3’
qPCRmTbp-R1	5’-TTCTTCACTCTTGGCTCCTGT-3’
mPigo exon6-F	5’-CATACCTACTCGGCTGCGAC-3’
mPigo exon6-R	5’-CGCAGGAACTGCTGGAACTC-3’
U6 promoter-U	5’-GAGGGCCTATTTCCCATGATTCC-3’
U6 promoter-L	5’-CCCAAGAAATTATTACTTTCTACGTCACG-3’
