# Supplementary material for: Establishment of mouse model of inherited PIGO deficiency and therapeutic potential of AAV-based gene therapy
Source: Nat Commun. 2022 Jun 3;13:3107. doi: 10.1038/s41467-022-30847-x (PMC9166810; doi:10.1038/s41467-022-30847-x)
Supplement: Supplementary file 2 — Reporting Summary [file 41467_2022_30847_MOESM2_ESM.pdf]

nature portfolio

Corresponding author(s): Yoshiko Murakami

Last updated by author(s): May 23, 2022

Reporting Summary

Nature Portfolio wishes to improve the reproducibility of the work that we publish. This form provides structure for consistency and transparency in reporting. For further information on Nature Portfolio policies, see our Editorial Policies and the Editorial Policy Checklist.

Statistics

For all statistical analyses, confirm that the following items are present in the figure legend, table legend, main text, or Methods section.

n/a	Confirmed	
☐	☒	The exact sample size (*n*) for each experimental group/condition, given as a discrete number and unit of measurement
☐	☒	A statement on whether measurements were taken from distinct samples or whether the same sample was measured repeatedly
☐	☒	The statistical test(s) used AND whether they are one- or two-sided<br>*Only common tests should be described solely by name; describe more complex techniques in the Methods section.*
☒	☐	A description of all covariates tested
☒	☐	A description of any assumptions or corrections, such as tests of normality and adjustment for multiple comparisons
☐	☒	A full description of the statistical parameters including central tendency (e.g. means) or other basic estimates (e.g. regression coefficient) AND variation (e.g. standard deviation) or associated estimates of uncertainty (e.g. confidence intervals)
☐	☒	For null hypothesis testing, the test statistic (e.g. *F*, *t*, *r*) with confidence intervals, effect sizes, degrees of freedom and *P* value noted<br>*Give P values as exact values whenever suitable.*
☒	☐	For Bayesian analysis, information on the choice of priors and Markov chain Monte Carlo settings
☒	☐	For hierarchical and complex designs, identification of the appropriate level for tests and full reporting of outcomes
☒	☐	Estimates of effect sizes (e.g. Cohen's *d*, Pearson's *r*), indicating how they were calculated

Our web collection on statistics for biologists contains articles on many of the points above.

Software and code

Policy information about availability of computer code

Data collection	Flowcytometry data was collected by MACSQuant Analyzer software (Miltenyi Biotec). EEG and video were recoded by Vital Recorder (Kissei Comtec). MRI data was collected by AVANCE II 500WB; (Bruker BioSpin).Western blot data was taken by ImageQuant™ LAS 4000 mini (Cytiva), BZ-X800 viewer (KEYENCE) was used to collect microscope image. Opera LX software (PerkinElmer) was used to collect confocal images, Mouse behavior analysis data was collected ANY-maze version 4.72 software (Stoelting Co). qPCR data was collected using StepOnePlus Real-Time PCR Systems (Thermofisher).
Data analysis	Flowcytometry data was analyzed by Flowjo software (Tommy digital). EEG with video tracking was analyzed by SleepSign (Kissei Comtec). Western blot images were analyzed by ImageQuant™ TL (Cytiva). Hybrid Cell Count (KEYENCE BZ-X800 analyzer)

For manuscripts utilizing custom algorithms or software that are central to the research but not yet described in published literature, software must be made available to editors and reviewers. We strongly encourage code deposition in a community repository (e.g. GitHub). See the Nature Portfolio guidelines for submitting code & software for further information.

Data

Policy information about availability of data

All manuscripts must include a data availability statement. This statement should provide the following information, where applicable:

- Accession codes, unique identifiers, or web links for publicly available datasets
- A description of any restrictions on data availability
- For clinical datasets or third party data, please ensure that the statement adheres to our policy

Full scan for western blots in Supplementary figure1 amd 4 are provided in the sourse data file. Raw data of the mouse analysis such as growth curves, blood tests and hanging tests in Figure 2 and 4 including 5'RACE of Pigo integration sites in Figure 6 and qPCR of ITR deriven Pigo expression are also provided in the source data file.

Human research participants

Policy information about studies involving human research participants and Sex and Gender in Research.

Reporting on sex and gender	*Use the terms sex (biological attribute) and gender (shaped by social and cultural circumstances) carefully in order to avoid confusing both terms. Indicate if findings apply to only one sex or gender; describe whether sex and gender were considered in study design whether sex and/or gender was determined based on self-reporting or assigned and methods used. Provide in the source data disaggregated sex and gender data where this information has been collected, and consent has been obtained for sharing of individual-level data; provide overall numbers in this Reporting Summary. Please state if this information has not been collected. Report sex- and gender-based analyses where performed, justify reasons for lack of sex- and gender-based analysis.*
Population characteristics	*Describe the covariate-relevant population characteristics of the human research participants (e.g. age, genotypic information, past and current diagnosis and treatment categories). If you filled out the behavioural & social sciences study design questions and have nothing to add here, write "See above."*
Recruitment	*Describe how participants were recruited. Outline any potential self-selection bias or other biases that may be present and how these are likely to impact results.*
Ethics oversight	*Identify the organization(s) that approved the study protocol.*

Note that full information on the approval of the study protocol must also be provided in the manuscript.

Field-specific reporting

Please select the one below that is the best fit for your research. If you are not sure, read the appropriate sections before making your selection.

☒ Life sciences ☐ Behavioural & social sciences ☐ Ecological, evolutionary & environmental sciences

For a reference copy of the document with all sections, see nature.com/documents/nr-reporting-summary-flat.pdf

Life sciences study design

All studies must disclose on these points even when the disclosure is negative.

Sample size	Sample size was chosen on the bases of prior experiences or literature (Human Molecular Genetics, 2020, Vol. 29, No. 7 1205–1217), so that the biological or technical viability would be sufficiently account for.
Data exclusions	No data was excluded.
Replication	All experiments have been successfully repeated with similar results for at least two to three times.
Randomization	For all the mouse studies, all the available and age matched KI or KIKO mice and AAV treated mice were used and the age matched wild type and hetero mice were randomly chosen for the experiments. For choosing mouse samples for MRI analysis and analysis of edited sites in the brain, we randomly chose two to four mice among the AAV treated mice. As for the analysis using knockout cells, we chose the representative clone among the several knockout clones and perform the repeated experiments using that clone.
Blinding	For the scoring of severity of tremor, one investigator was blinded to pick up the mouse from the cages for the observation and determined the score together with another blinded investigator. After finishing observation of all the mice in the cage, genotype of the mice were checked. As for the seizure score, the blinded investigator watched the PTZ treated mice and determined the score.

Reporting for specific materials, systems and methods

We require information from authors about some types of materials, experimental systems and methods used in many studies. Here, indicate whether each material, system or method listed is relevant to your study. If you are not sure if a list item applies to your research, read the appropriate section before selecting a response.

Materials & experimental systems

n/a	Involved in the study
☐	☒ Antibodies
☐	☒ Eukaryotic cell lines
☒	☐ Palaeontology and archaeology
☐	☒ Animals and other organisms
☒	☐ Clinical data
☒	☐ Dual use research of concern

Methods

n/a	Involved in the study
☒	☐ ChIP-seq
☐	☒ Flow cytometry
☐	☒ MRI-based neuroimaging

Antibodies

Antibodies used	rat anti-mGr-1(Biolegend), goat anti-human contactin-1(R&D systems), rat anti-mouse Thy1(G7, Biolegend), rat anti-MBP (MCA409S,BioRad), mouse anti-GAPDH (AM4300,Invitrogen), O4(MAB1326 R&D), anti-Contactin2 (ab133498 abcam), anti-Synapsin (106103, SYnaptic System), goat anti-mouse IgM Alexa488 (A21042, Invitrogen), goat anti-rat IgG Rhodamin (AP183R, CHemicon), donkey anti-rabbit IgG FITC (711-096-152, Jackson), anti-Nestin (ab6142, abcam), anti-β-tubulin (ab18207, abcam), anti-MAP2 (ab5392,abcam), anti-NeuN (ab17787, abcam), anti-Synapsin (106 004,Synaptic systems), anti-Tau-1 (MAB3420, Chemicon), goat anti-mouse IgG-Alexa488 (A11029, Life Technologies), goat anti-chiken IgY-Alexa555 (Life Technologies), goat anti-rabbit-alexa647 (Life Technologies), goat anti-guinea pig IgG Alexa488 (Life Technologies), Biotin-rat anti-mouse CD24 monoclonal M1/69 BD 553260 lot 0000042453) anti-hCD59 (5H8)
Validation	For the antibodies for GPI anchored proteins, such as Gr-1, contactin-1, 2, Thy-1, CD24, CD59, they were validated by staining of GPI knockout cells compared with wild type cells in FACS, WB, and IHC. The other antibodies were validated using the isotype control. Detailed information could be found on the manufactures' web site.

Eukaryotic cell lines

Policy information about cell lines and Sex and Gender in Research

Cell line source(s)	HEK293 cells(ATCC CRL-1573) Neuro2a cells (ATCC CCL-131), NIH/3T3(ATCC CRL-1658)
Authentication	All the cell lines were maintained in the lab and not authenticated for this study.
Mycoplasma contamination	Cell lines were not tested for mycoplasma.
Commonly misidentified lines (See ICLAC register)	No commonly misidentified lines was used.

Animals and other research organisms

Policy information about studies involving animals; ARRIVE guidelines recommended for reporting animal research, and Sex and Gender in Research

Laboratory animals	All mice are C57BL/6 background; Mice were maintained in SPFunder a 12-hour light/dark cycle and given free access to food and water. Temperature and humidity is within the recommended range(20~24° 40~60%)
Wild animals	No wild animals was used.
Reporting on sex	Figure1f, FACS 4months wild, KI hetero, homo, KI/KO all males, 1g, ALP 4months wild,female5, male8; KI hetero,female9,male6; homo,female9, male15; KI/KO all males, Figure 4a FACS 4months homo, female2, male2; AAVtreated,female4, male3; KI/KO, all males; AAVtreated all males; 4b,ALP 4months female2, male2; AAV treated female5, male4; wild, female3, male2; KI/KO female5, male3; AAV treated female2, male1; wild female3, male2, 4g,hanging test 5months homo, female4, male3 AAVtreated,female3, male3, Figure 7c, FACS, 4months, KI/KO,all males; HITItreated all males, Donor treated, female2, male2; wild, all females, 7d, ALP 4months, KI/KO, female3, male3; HITItreated, female1, male2; Donor treated, female2, male2; wild, female3, male2; 7e, Hamging test & tremor, 5months, HITItreated, female2, male1; Donor treated female3, male3,
Field-collected samples	This study did not involve field-collected samples.
Ethics oversight	All animal procedures were approved by the Animal Care and Use Committee of the Research Institute for Microbial Diseases, Osaka University, Japan.

Note that full information on the approval of the study protocol must also be provided in the manuscript.

Flow Cytometry

Plots

Confirm that:

- [x] The axis labels state the marker and fluorochrome used (e.g. CD4-FITC).
- [x] The axis scales are clearly visible. Include numbers along axes only for bottom left plot of group (a 'group' is an analysis of identical markers).
- [x] All plots are contour plots with outliers or pseudocolor plots.
- [x] A numerical value for number of cells or percentage (with statistics) is provided.

Methodology

Sample preparation	Cell lines and mice blood were suspended with PBS containing 1% BSA and stained with fluorescent labeled antibodies. Mouse blood cells were pretreated with ACK buffer to lyse RBC.
Instrument	MACSQuant Analyzer (Miltenyi Biotec)
Software	Flowjo software (Tommy Digital)
Cell population abundance	At least 10000 live cells in the target gate were analyzed.
Gating strategy	Always put the isotype control staining for a negative population. Cell lines were gated with FSC/SSC for live cells. As for the mouse blood cells, lineage specific markers were used for gating.

- [ ] Tick this box to confirm that a figure exemplifying the gating strategy is provided in the Supplementary Information.

Magnetic resonance imaging

Experimental design

Design type	No task, only for structural imaging
Design specifications	not fMRI
Behavioral performance measures	not fMRI

Acquisition

Imaging type(s)	No task, only for structural imaging
Field strength	11.7T
Sequence & imaging parameters	Rapid Acquisition with Relaxation Enhancement (RARE) sequence, field of view = 20 mm x 20 mm, matrix size = 256 x 256, slice thickness = 300 µm, sagittal and coronal images, repetition time [TR] = 6000 ms, echo time [TE] = 37.6 ms, flip angle = 180 degree, number of averages = 12, acquisition time = 19 min.
Area of acquisition	whole brain

Diffusion MRI ☐ Used ☐ Not used

Preprocessing

Preprocessing software	n/a
Normalization	none
Normalization template	none
Noise and artifact removal	none
Volume censoring	none

Statistical modeling & inference

Model type and settings	none

Effect(s) tested	none

Specify type of analysis: ☐ Whole brain ☐ ROI-based ☐ Both

Statistic type for inference (See Eklund et al. 2016)	none
Correction	none

Models & analysis

n/a	Involved in the study
☒	☐ Functional and/or effective connectivity
☒	☐ Graph analysis
☒	☐ Multivariate modeling or predictive analysis
