# Supplementary material for: Establishment of mouse model of inherited PIGO deficiency and therapeutic potential of AAV-based gene therapy
Source: Nat Commun. 2022 Jun 3;13:3107. doi: 10.1038/s41467-022-30847-x (PMC9166810; doi:10.1038/s41467-022-30847-x)
Supplement: Supplementary file 4 — Description of Additional Supplementary Files [file 41467_2022_30847_MOESM4_ESM.pdf]

Title: Supplementary Movie 1.

Description: *Pigo*$^{b/b}$mouse showing tremor of score 1 (weakest of five scores).

Title: Supplementary Movie 2.

Description: *Pigo*$^{b/b}$mouse showing tremor of score 5 (strongest of five scores).

Title: Supplementary Movie 3.

Description: Comparison of the HITI-TE-treated *Pigo*$^{b/b}$ mouse and the untreated *Pigo*$^{b/b}$ mouse. At the beginning of the video, the untreated *Pigo*$^{b/b}$ mouse on the right side exhibited ataxic gate dragging of hindlimbs and severe tremors. The untreated *Pigo*$^{b/b}$ mouse tended to stay in the same place. The HITI-TE-treated *Pigo*$^{b/b}$ mouse on the left side had no apparent tremor and smoothly walked a longer distance than the untreated mouse.
